# Molten salt-directed catalytic synthesis of room-temperature ferromagnetic transition-metal nitride nanosheets

**DOI:** 10.1039/d5ra08017g

**Published:** 2026-01-19

**Authors:** Wei Hu, Ruiqi Liu, Yunlai Zhu, Chao Wang

**Affiliations:** a School of Integrated Circuits, Anhui University 111 JiuLong Road Hefei Anhui 230601 P. R. China huwei65@ahu.edu.cn; b Anhui Provincial High-performance Integrated Circuit Engineering Research Center 111 JiuLong Road Hefei Anhui 230601 P. R. China; c National Synchrotron Radiation Laboratory, University of Science and Technology of China 96 JinZhai Road Hefei Anhui 230029 P. R. China chaowng@ustc.edu.cn; d School of Nuclear Science and Technology, University of Science and Technology of China 96 JinZhai Road Hefei Anhui 230029 P. R. China

## Abstract

The next generation of low-power electronic/spintronic devices based on two-dimensional (2D) magnetic materials has great application prospects. However, so far, the numbers of intrinsic room-temperature magnetic 2D materials is limited. Here, we report a simple and effective three-step molten salt-directed catalytic method for obtaining intrinsic room-temperature ferromagnetic gamma-Mo_2_N (γ-Mo_2_N) nanosheets. The γ-Mo_2_N nanosheets with high crystallization and ultra-thin 2D layered structure characteristics show obvious ferromagnetism and Curie temperature (*T*_c_) up to about 360 K. Meanwhile, ICP-AES measurement excludes the possibility of ferromagnetic impurities. Extensive synchrotron radiation X-ray absorption fine structure (XAFS) spectra characterizations confirm the bonding configuration of Mo–N coordination around Mo atoms as well as the structural stability of the samples. Detailed spin-polarized density functional theory (DFT) calculations reveal that ferromagnetism of γ-Mo_2_N nanosheets is mainly contributed by 4d electrons of Mo atoms with itinerant electron characteristics. This work highlights γ-Mo_2_N nanosheets as a promising intrinsic room-temperature ferromagnetic material for the development of spintronics or spin-based electronics.

## Abstract 2

We report a facile molten-salt strategy to synthesize ultrathin γ-Mo_2_N nanosheets, which exhibit intrinsic room-temperature ferromagnetism. This work establishes transition metal nitrides as a new class of 2D magnetic materials for spintronics.

## Graphical abstract

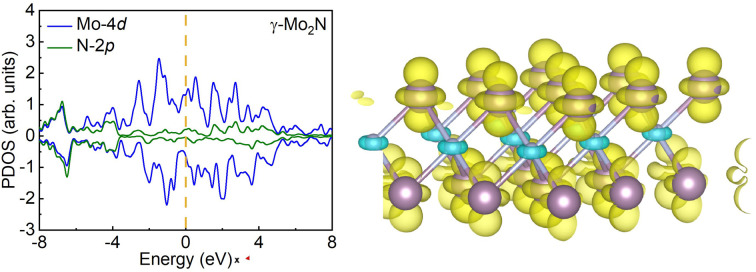

## Introduction

Since the discovery of graphene, two-dimensional (2D) materials have shown many novel physical properties different from their bulk phase crystals due to the low dimensional characteristics.^1–3^ For example, graphene has excellent heat and electron conduction properties, as well as the unique electronic structure of the dirac cone.^4,5^ In the past few decades, various strategies have been developed to synthesize low-dimensional materials, resulting in some new 2D structures and a wide range of practical performance. Among these performances, nanoelectronics, especially spin electronics, is one of the most important application fields. However, most 2D materials, such as graphene, hexagonal boron nitride (BN), and transition metal chalcogenides (TMDs), are inherently non-magnetic. Therefore, to make them suitable for spin-related applications, various means of spin injection or induction are usually required.^6–8^ However, the magnetic response introduced by these methods is local and non-intrinsic, and it is difficult to achieve flexible external field control. The search for novel 2D materials with intrinsic and room-temperature ferromagnetism is of great significance for the further development of spin-dependent low-dimensional electronic devices. In 2017, Zhang *et al.* and Xu *et al.* discovered that the intrinsic long-range ferromagnetic response in pristine Cr_2_Ge_2_Te_6_ atomic layers and monolayer CrI_3_ at low temperature,^9,10^ respectively, expands the scope of 2D magnets. Thereafter, itinerant ferromagnetism in monolayer Fe_3_GeTe_2_ was also found with Curie temperature (*T*_c_) to be less than 205 K.^11^ However, the environmental instability and low *T*_c_ of these 2D van der Waals (vdW) ferromagnets limit their practical applications. Therefore, it is necessary to search for new 2D intrinsic ferromagnetic systems that can work stably at room temperature.

Recently, some 2D structures based on transition metal nitrides (TMNs) have shown many excellent properties, such as semi-metallicity, piezoelectric, energy storage, and catalytic performance, greatly promoting the development of existing 2D materials.^12–14^ Wherein the TM can provide atomic magnetic moment, giving a possibility for TMNs to obtain macroscopic magnetism. Meanwhile, the strong hybridization between the d orbitals of TM and p orbitals of N can give TMNs excellent stability.^15^ Theoretical calculations have predicted that a series of TMNs nanosheets exhibit intrinsic ferromagnetism with relatively high *T*_c_.^16–19^ For example, CrN is a typical TMN material, and studies have shown that there may be ferromagnetic ordering in its 2D lattice;^19^ the pentagonal MnN_2_ nanosheets are considered a type of ferromagnet, with theoretically calculated *T*_c_ up to 956 K.^17^ Experimentally, Gogotsi *et al.* reported the intrinsic magnetic behavior in 2D Mn_3_N_2_ flakes even at 300 K;^20^ Yao *et al.* designed spintronic devices based on the ferromagnetic YN_2_ monolayer, and found that they exhibit dual spin filtering and dual spin diode as well as the spin Seebeck effect when a bias voltage is applied.^21^ However, compared to other similar 2D materials such as transition metal oxides (TMO) and TMDs, researches on TMNs are much less. Part of the reason is the high stability of N_2_ molecules, which makes it relatively difficult to synthesize TMNs, especially 2D TMNs.^22,23^ For example, the existence of 1036 strippable layered materials has been predicted through high-throughput calculations, but only one of them is vdW layered 2D TMNs;^24^ at the same time, the experimental results on the magnetism of TMNs are far less than theoretical calculations. Traditional methods for synthesizing 2D materials, such as chemical vapor deposition, liquid exfoliation, and mechanical exfoliation are limited in the preparation of 2D TMNs. For the growth of 2D TMNs, the most effective method is to use high pressure to prevent the diffusion of N atoms from the metal lattice outward at high temperatures.^25,26^ However, this method is costly and dangerous, so it is necessary to develop feasible preparation methods under atmospheric pressure. In recent years, environmentally friendly and efficient molten salt-assisted methods have played a crucial role in the preparation of 2D materials.^27–31^ For example, Jin *et al.* proposed a preparation route of molten alkali salt-directed catalysis to synthesize 2D layered TMNs under atmospheric pressure.^28^ Among them, alkali metal salts act as catalysts rather than conventional reactants, promoting the growth of 2D TMNs by lowering the melting point of metal oxide precursors, further reducing the formation energy, and stabilizing the layered structure. Motivated by the above considerations, we anticipate that 2D TMNs with room-temperature ferromagnetism could be prepared by the molten salt-directed catalytic method, a general strategy that also holds great promise for synthesizing a broader family of low-dimensional magnetic material beyond nitrides.

In this work, we investigate the magnetic properties of 2D TMNs experimentally and theoretically. Using a three-step process of molten salt-directed catalytic method, we successfully synthesized single-phase and highly crystalline 2D layered γ-Mo_2_N nanosheets. This synthesis strategy represents a significant simplification compared to previously reported high-pressure or complex vapor-deposition techniques for TMNs. The products exhibit intrinsic ferromagnetic orderings with *T*_c_ up to about 360 K, which not only confirms the existence of room-temperature ferromagnetism in 2D TMNs but also surpasses the *T*_c_ of many other prominent 2D magnets, such as CrI_3_ and Fe_3_GeTe_2_. Detailed and in-depth X-ray spectroscopy characterization technologies have confirmed the bonding configuration of Mo–N coordination around Mo atoms, as well as the structural stability of the samples. Electronic structure calculations uncover that strong hybridization between d orbitals of Mo atoms and p orbitals of N endow 2D γ-Mo_2_N with good stability, and intrinsic room-temperature ferromagnetic couplings are mainly attributed to the contributions of Mo 4d electrons. Our findings provide a new hint for the synthesis of 2D TMNs with intrinsic room-temperature ferromagnetism and promise the potential applications of TMNs in next-generation spintronics or spin-based electronics.

## Results

### Analysis of sample morphology and structure

Most metals/metal oxides have a very high melting point, mixing them with alkali metal salts can significantly lower the melting points; further, in the molten state, the metal precursors can melt into monomers, so it has higher reactivity and faster reaction rate.^32^ Using a three-step process of molten salt-directed catalytic method,^28^ the γ-Mo_2_N nanosheets were prepared. Specific steps are as follows: (i) metal oxide powders and alkali metal salts were mixed through ball milling; (ii) then the mixtures were annealed at 650 °C in an Ar atmosphere containing 5% NH_3_; (iii) finally, the products were ultrasonic washed in deionized water, and freeze-dried to obtain layered TMNs nanosheets. The structure and morphology of synthesized products are analyzed in detail below.

**Fig. 1:**
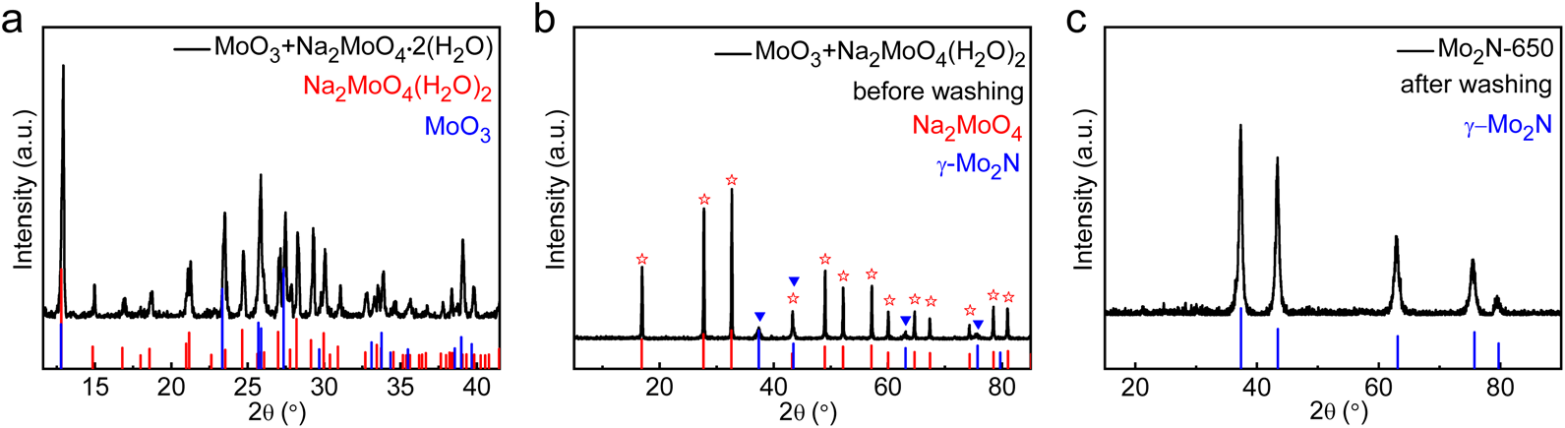
(a) XRD pattern of MoO_3_ and Na_2_MoO_4_·2H_2_O precursor powders mixed by ball milling. (b) XRD pattern of the mixed precursors after annealing at 650 °C in an Ar atmosphere containing 5% NH_3_ for 5 h (before ultrasonic cleaning). (c) XRD pattern of the final products after ultrasonic cleaning and lyophilization.

Fig. 1a shows the X-ray diffraction (XRD) pattern of the mixed precursor powders (Na_2_MoO_4_·2H_2_O and MoO_3_) measured after the first step of ball milling. The diffraction peaks of two precursors, Na_2_MoO_4_ 2H_2_O (JCPDS No. 70-1710) and MoO_3_ (JCPDS No. 35-0609), can be observed in the figure, no additional phases were generated during the milling process. To further illustrate the chemical composition of prepared samples, the XRD patterns of annealed products before (the second step) and after (the third step) ultrasonic washing with deionized water were also measured. As shown in Fig. 1b, two phases of γ-Mo_2_N (JCPDS No. 25-1366) and Na_2_MoO_4_ can be observed in the products before washing. This indicates that after annealing with NH_3_, a new phase was formed, but the composition of alkali metal salts did not change, indicating precursor alkali metal salts played a catalytic role in the reaction.^28^ Further removal of alkali metal salts through deionized water washing resulted in the final product phase as shown in Fig. 1c. There are five obvious diffraction peaks corresponding to the (111), (200), (220), (311), and (222) planes of γ-Mo_2_N.^33^ Accordingly, the lattice parameters are *a* = *b* = 2.88 Å, with angles *α* = *β* = 90°, *γ* = 120°. The corresponding Wyckoff positions are Mo at 6c (0, 0, 0.242891) and N at 3a (0, 0, 0). The XRD results show that we have obtained a pure gamma-phase Mo_2_N through the three-step experimental methods.

**Fig. 2:**
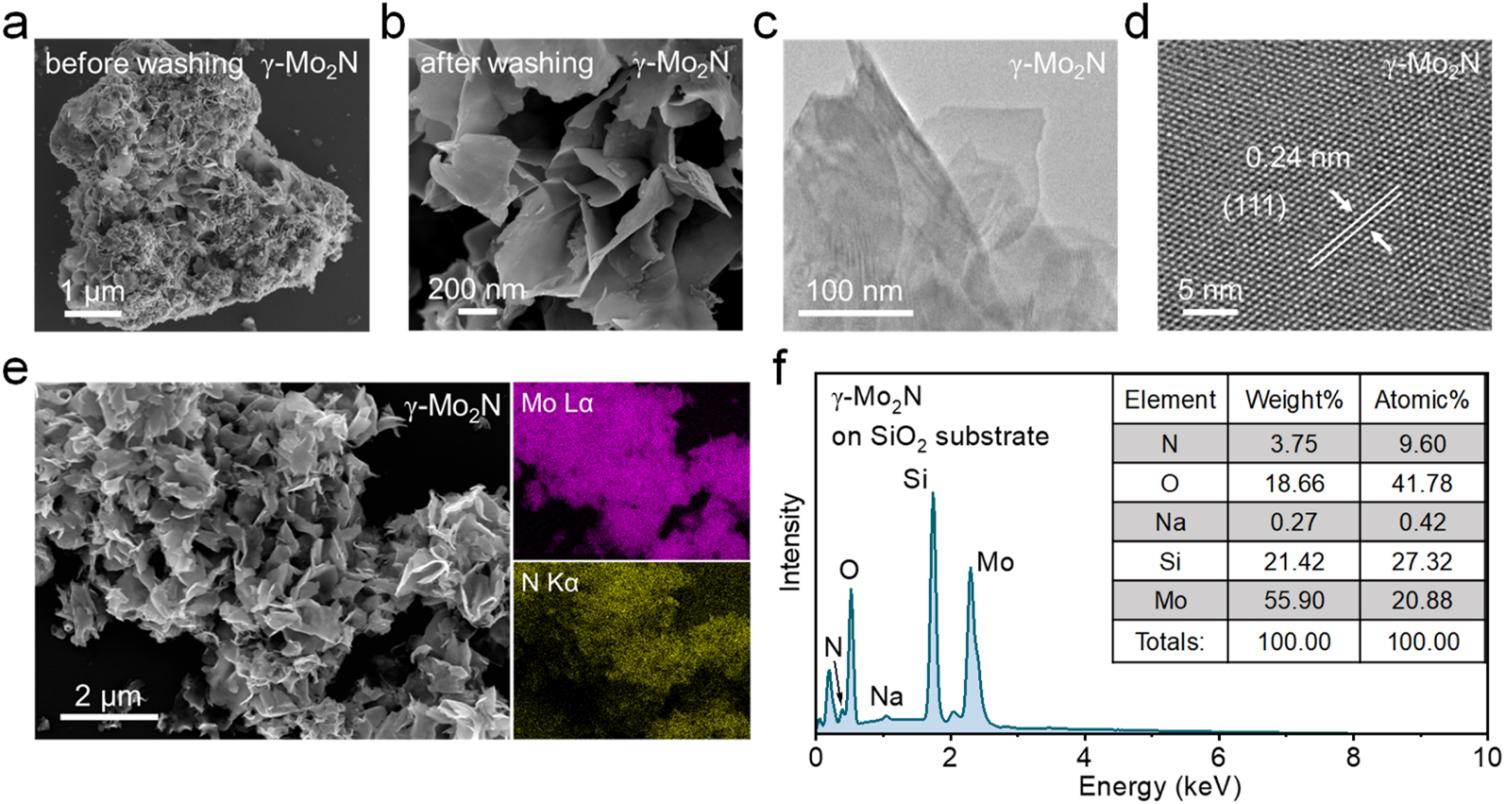
(a) Low-resolution SEM image of synthesized γ-Mo_2_N before washing. (b) High-resolution SEM, (c) TEM, (d) HRTEM, (e) elemental mapping, and (f) EDS of synthesized γ-Mo_2_N after washing.

The morphology and atomic structure of γ-Mo_2_N can be directly observed from scanning electron microscopy (SEM) and transmission electron microscopy (TEM). Fig. 2a and b shows the SEM images of synthesized γ-Mo_2_N before and after washing with deionized water. Compared with the samples before washing, the 2D morphologies of γ-Mo_2_N are well preserved (Fig. 2b), indicating that the molybdenum-based TMNs have good structural stability. Notably, the samples exhibit the form of atomically thin 2D structures rather than bulk particles before washing. The corresponding low-resolution TEM result of γ-Mo_2_N after washing is shown in Fig. 2c, the combination of nanoscale lateral dimensions with high transparency to the electron beam is a physical property characteristic of ultrathin, 2D nanosheets. The atomic structure was detected by high-resolution TEM (HRTEM) (Fig. 2d), which shows the well-crystalline nature of the γ-Mo_2_N nanosheets with an interplanar spacing of ∼0.24 nm, corresponding to the (111) planes of γ-Mo_2_N crystal. The energy dispersive X-ray spectroscopy (EDS) mapping images (Fig. 2e) indicate the homogeneous distribution of Mo and N elements over the entire flakes, and the atomic ratio of Mo and N in γ-Mo_2_N nanosheets is about 2 (Fig. 2f), which aligns with the stoichiometry of γ-Mo_2_N. It is noted that the absolute atomic percentages are influenced by the SiO_2_ substrate used for this measurement.

**Fig. 3:**
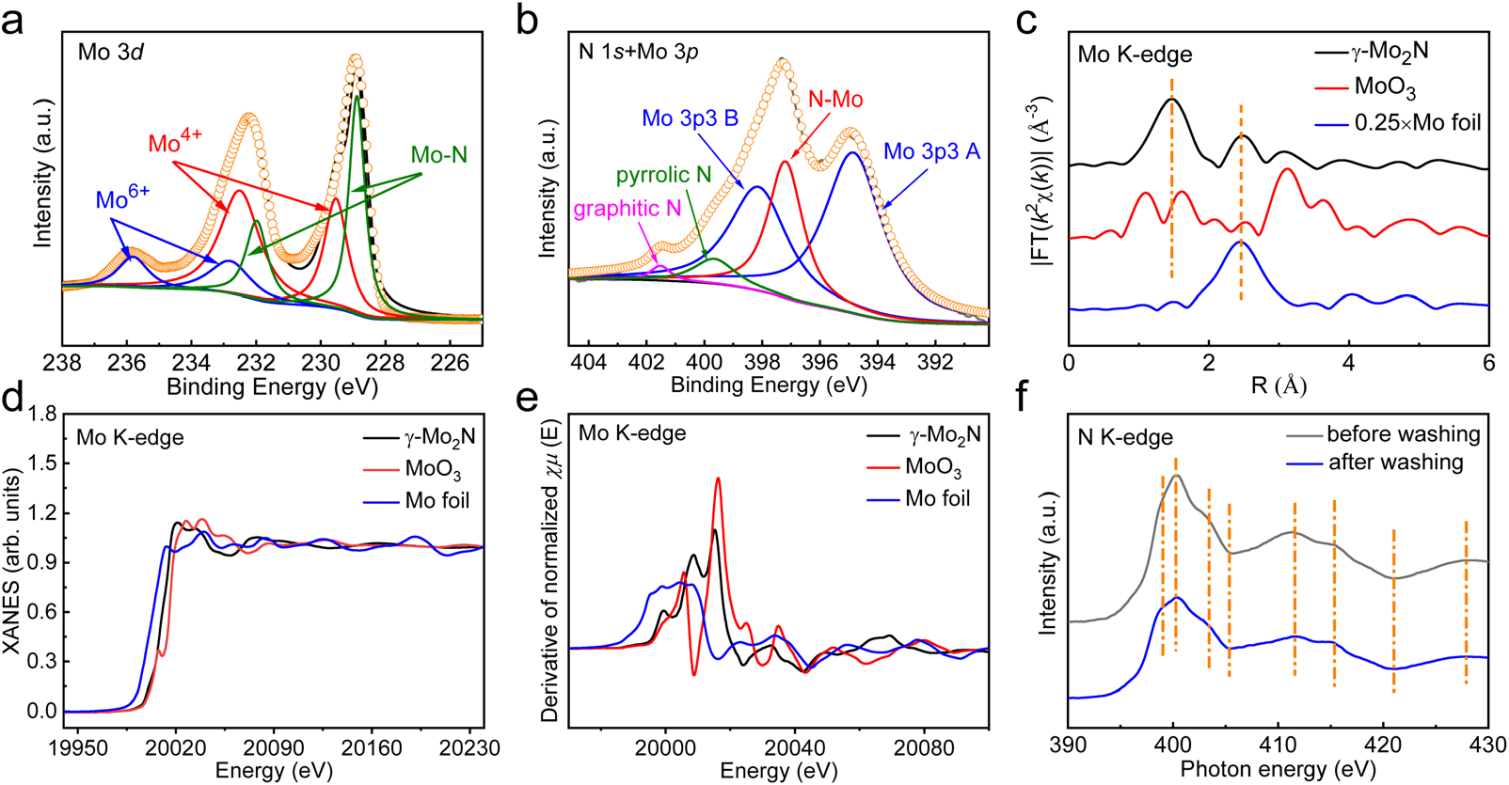
(a) High-resolution Mo 3d and (b) N 1s + Mo 3p XPS spectra of γ-Mo_2_N nanosheets. (c) FT Mo K-edge EXAFS, (d) Mo K-edge XANES, and (e) first-derivative Mo K-edge XANES curves of γ-Mo_2_N nanosheets and reference samples. (f) N K-edge XANES spectra of γ-Mo_2_N nanosheets before and after washing.

The bonding configurations of γ-Mo_2_N nanosheets were characterized by the X-ray photoelectron spectroscopy (XPS), Mo K-edge extended X-ray absorption fine structure (EXAFS), and N K-edge X-ray absorption near edge structure (XANES) spectra. There are six peaks in the high-resolution Mo 3d spectrum (Fig. 3a), the peaks at about 299.5 eV and 232.5 eV are from the contribution of Mo^4+^ signals, and the peaks at about 232.8 eV and 235.8 eV are due to the contribution of Mo^6+^ signals.^34,35^ In addition, the peaks of 228.9 eV and 232.0 eV correspond to the characteristic peaks of Mo–N bonds in γ-Mo_2_N.^34–36^ It is worth noting that, only a phase of γ-Mo_2_N can be detected in the XRD results above, which inferred that the related substances of Mo^4+^ and Mo^6+^ are amorphous. The existence of these oxides is related to the oxidation of the nitride surface, which can be observed in many Mo-based carbides or nitrides.^37–39^ The high-resolution N 1s XPS spectrum of γ-Mo_2_N nanosheets (Fig. 3b) can be deconvoluted into three peaks at 401.5 eV, 399.7 eV and 397.2 eV, which are ascribed to graphitic N, pyrrolic-N and Mo–N, respectively.^8,35^ In addition, the peaks at 398.2 eV and 394.9 eV are attributed to Mo 3p XPS, which overlap with the N 1s peaks.^35^ The Fourier-transformed (FT) Mo K-edge EXAFS spectra of γ-Mo_2_N nanosheets and related reference samples (MoO_3_ and Mo foils) are shown in Fig. 3c. Among them, γ-Mo_2_N nanosheets exhibit two obvious absorption peaks at the positions of 1.45 Å and 2.46 Å, which can be assigned to the scattering of Mo–N and Mo–Mo coordination, respectively, consistent with the coordination shell information of γ-Mo_2_N reported in previous literatures.^40,41^ Two representative peaks of the MoO_3_ sample below 2 Å can be indexed to the Mo=O (1.09 Å) and Mo–O (1.62 Å) bonds.^42^ Meanwhile, the Mo K-edge XANES (Fig. 3d) and first derivative spectrum (Fig. 3e) for γ-Mo_2_N nanosheets are significantly different from that of the reference samples, suggesting the positive valence state of Mo atoms (between the two samples of Mo foil and MoO_3_) in γ-Mo_2_N nanosheets and further excluding the possibility of metallic or/and oxide clusters of Mo. Moreover, considering that the only constant element during the washing process is N, we conducted synchrotron XANES to observe the N K-edge absorption spectrum of samples before and after washing. As shown in Fig. 3f, the N K-edge XANES did not change its states, further demonstrating the structural stability of 2D γ-Mo_2_N even after salts removal. All the above experimental results lead us to conclude that we have successfully prepared 2D γ-Mo_2_N nanosheets through a three-step molten salt-directed catalytic method.

### Ferromagnetism of synthesized γ-Mo_2_N nanosheets

**Fig. 4:**
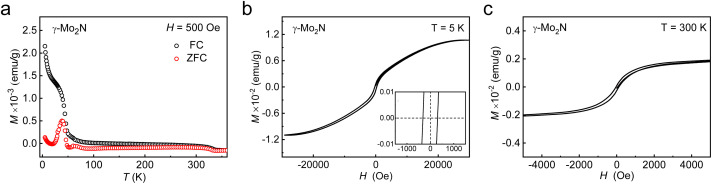
(a) Temperature dependence of FC and ZFC magnetization (*M*–*T*) curves for γ-Mo_2_N nanosheets. *M*–*H* curves for γ-Mo_2_N nanosheets at (b) 5 K and (c) 300 K after background deduction.

To clarify the magnetic properties of synthesized γ-Mo_2_N nanosheets, we measured the magnetization with temperature (*M*–*T*) curves in the field cooling (FC) and zero field cooling (ZFC) modes and field-dependent magnetization (*M*–*H*) curves. The *M*–*T* curves of γ-Mo_2_N nanosheets under an applied field of 500 Oe are given in Fig. 4a with obvious thermo-magnetic irreversibility (a bifurcation between the FC and ZFC modes) above room temperature (up to ∼360 K), indicating that γ-Mo_2_N nanosheets are ferromagnetic.^8^ In addition, the magnetization difference between FC and ZFC modes (ΔM, Δ*M* = *M*_FC_ − *M*_ZFC_) exhibits positive values throughout the entire temperature range, thus ruling out the possibility of spin glass effect and superparamagnetism in the samples.^8^ The negative magnetization observed at 50 K under ZFC mode and around 300 K under FC case does not originate from the intrinsic magnetic response of γ-Mo_2_N sample. Instead, this feature arises from a diamagnetic background contributed by the sample holder and packing materials used for powder measurements, which has also been observed in previous studies on other material systems.^43,44^ The *M*–*H* curve of γ-Mo_2_N nanosheets at 5 K (Fig. 4b) exhibits typical hysteresis loop characteristics and coercivity is about 310 Oe. The well-defined hysteresis loop can also be observed in the *M*–*H* curve at 300 K (Fig. 4c), which indicates that the ferromagnetism of γ-Mo_2_N nanosheets can be stabilized to room temperature. From Fig. 4b and c, it can be seen that after subtracting the diamagnetic background in the *M*–*H* curves at 5 K and 300 K, we obtained typical ferromagnetic hysteresis loops, in that the magnetization remains positive under positive magnetic fields. Besides, ferromagnetic transition metal elements such as Fe, Co, and Ni were not detected through ICP-AES measurements, which ruled out the possibility of introducing magnetic impurities during the preparation process and demonstrated that the ferromagnetism of γ-Mo_2_N nanosheets is intrinsic.

### Origin of the ferromagnetism in γ-Mo_2_N nanosheets

**Fig. 5:**
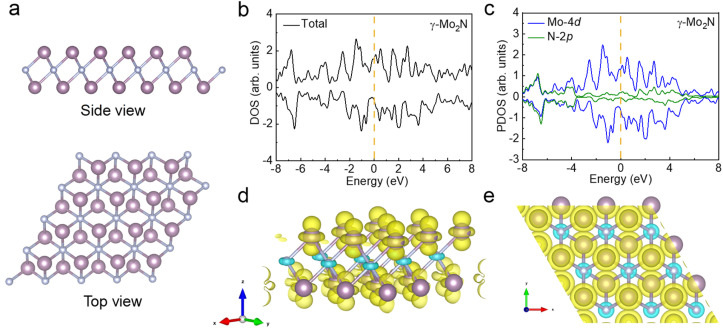
(a) Side and top views for the 2D γ-Mo_2_N structure, the purple and gray balls represent Mo and N atoms, respectively. DFT calculated (b) TDOS and (c) PDOS for 2D γ-Mo_2_N structure. The dashed lines in (b) and (c) indicate the Fermi level. (d) Side and (e) top views for the spin density (ρ↑–ρ↓) distribution of 2D γ-Mo_2_N structure, the yellow and blue isosurfaces indicate the spin-up and spin-down states, respectively.

To gain an in-depth understanding of the origin for the room-temperature ferromagnetism in γ-Mo_2_N nanosheets, we employed spin-polarized density functional theory (DFT) calculations (see details in the Methods section). The calculations in this work employed a single-layer structure of γ-Mo_2_N, based on experimental evidence from TEM characterization showing electron-transparent nanosheets. This approach allows us to investigate the material in the two-dimensional limit and establish the fundamental properties of the system. The monolayer model serves as the minimal theoretical unit that captures the essential physics of the 2D system, free from interlayer interactions. This computational strategy is particularly suited for identifying the intrinsic origin of emergent properties in the ideal 2D limit. The 2D structure diagrams, total densities of states (TDOS), projected densities of states (PDOS), and spin density (ρ↑–ρ↓) distribution are presented in Fig. 5a–e, respectively. 2D γ-Mo_2_N structures are obtained by extracting a stoichiometric layer from the bulk γ-Mo_2_N crystal. Fig. 5a illustrates the schematic representation of the 2D γ-Mo_2_N structure with top and side views, in which purple and gray balls represent Mo and N atoms, respectively. The TDOS result of the γ-Mo_2_N structure in Fig. 5b shows unequal spin-up and spin-down branches, indicating the presence of magnetic states. Further, detailed PDOS analyses are given in Fig. 5c, where there is strong hybridization between the d orbitals of Mo atoms and p orbitals of N, ensuring the stability of the sample structure.^15^ Meanwhile, it can be seen that Mo-4d and N-2p orbitals are both spin-polarized with asymmetry between spin-up and spin-down states, and induced magnetic moments are mainly attributed to the contribution of 4d electrons for Mo atoms, with a value of approximately 0.3 µ_B_/Mo, while the induced moment on the N atoms is negligible (−0.06 µ_B_/N). The spin density (ρ↑–ρ↓) distributions as shown in Fig. 5d and e also indicate that spins in the 2D γ-Mo_2_N structure are mainly located at Mo atoms, which is the same as the result of PDOS. In addition, the DOS results confirm that 2D Mo_2_N is metallic. Notably, there is a significant distribution of Mo-4d electron bands near the Fermi level. A pronounced spin-polarization is observed, with the density of states being predominantly contributed by the spin-up channels, leading to a strong asymmetry between the spin-up and spin-down components. These indicate that d electrons in this part are not only relatively delocalized but also highly spin-polarized, reflecting the ferromagnetic characteristics of itinerant electrons.^45^ While real samples may contain few-layer regions, the single-layer calculation provides crucial insights into the fundamental behavior of the material. The consistency between our computational results and experimental measurements validates this approach for understanding the electronic origin of ferromagnetism in 2D γ-Mo_2_N.

In addition, we constructed a 2 × 2 × 1 supercell of monolayer γ-Mo_2_N (Fig. S1, as shown in the SI), which contains 8 inequivalent Mo atoms. This supercell allows us to model a variety of magnetic configurations, including the ferromagnetic (FM) state and several antiferromagnetic (AFM) states by flipping the spins of different Mo sublattices. The calculated total energies for these configurations are summarized in the Table S1 (which has also been added to the SI). The key finding is that the FM state has the lowest energy, confirming it as the magnetic ground state, which is fully consistent with our experimental observations.

## Discussion

In summary, we have explored the magnetism of 2D transition metal nitrides, using a three-step molten salt-directed catalytic method to successfully synthesize highly crystalline γ-Mo_2_N nanosheets with obvious ferromagnetism of Curie temperature up to 360 K. Detailed studies of structural, electronic, and magnetic properties suggest that strong hybridization between d orbitals of Mo and p orbitals of N endow 2D γ-Mo_2_N with good stability, and the observed ferromagnetism is mainly contributed by the 4d electrons for Mo atoms. The delocalized and highly spin-polarized d electrons near the Fermi level meet the characteristics of itinerant electrons. The γ-Mo_2_N nanosheets with intrinsic room-temperature ferromagnetism prepared in this work provide a possibility for the application of transition metal nitrides in the next generation of spintronics. Furthermore, the proposed molten salt-directed catalytic approach holds significant promise for being extended to synthesize other families of 2D magnetic materials, such as transition metal carbides/sulfides,^46^ thereby opening new avenues for exploring novel magnetism in low-dimensional systems.

## Methods

### Synthesis of γ-Mo_2_N nanosheets

First, 2.42 g Na_2_MoO_4_·2H_2_O and 1.44 g MoO_3_ powders were mixed *via* ball milling with a rotation speed of 400 revolution per minute (rpm) for 40 min. Then, 200 mg of these mixtures were spread flatly into a porcelain boat. It is worth noting that the thickness of accumulation should not be too large, otherwise the reaction will be incomplete. The porcelain boat was then placed in the center of a quartz tube furnace and annealed for 5 h at 650 °C (heating rate 1°C min^−1^) in an Ar atmosphere containing 5% NH_3_ (75 sccm). After annealing, the samples were ultrasonic washed in deionized water, centrifuged to remove the salt solution, and finally freeze-dried to obtain γ-Mo_2_N nanosheets.

### DFT calculation details

The crystal structure of monolayer γ-Mo_2_N was fully optimized. The theoretically optimized lattice parameters are *a* = *b* = 2.73 Å, with angles *α* = *β* = 90°, *γ* = 120°. The spin-polarized DFT calculations with projector augmented wave (PAW) were performed with the Quantum Espresso software package.^47^ The exchange-correlation was described with the generalized gradient approximation (GGA) in the Perdew–Burke–Ernzerhof (PBE)^48^ parametrization. The kinetic energy cutoffs for the plane wave and electron density were 75 Ry and 500 Ry. LDA + U correction with *U* = 4.0 eV was applied for the Mo d orbitals. The DFT-D3 scheme^49^ was employed to process the long-range van der Waals interaction. The Brillouin zone was integrated in 5 × 5 × 1 k-grid and 15 × 15 × 1 k-grid for structure optimizations and electronic structure calculations, respectively. The convergence criteria for SCF calculations and structure optimizations were 1.0 × 10^−3^ meV/atom and 0.05 eV Å^−1^. A vacuum layer of 20 Å was set along *z*-axis to avoid interlayer interaction.

### Characterization

TEM and EDS measurements were performed on a JEM-2100 F field transmission electron microscope with an acceleration voltage of 200 kV. The HRTEM was tested on a JEOL JEMARF200 F TEM/STEM with spherical error corrector. SEM measurements were performed on a scanning transmission electron microscope (SEM, JSM-6700 F, 5 kV). The XRD spectra were collected under the Cu *K*_α_ radiation (*λ* = 1.54178 Å) of the Philips X ’Pert Pro super diffractometer. XPS was obtained on ESCALAB MKII using Mg Kα (hν = 1253.6 eV) as excitation source. The Mo K-edge EXAFS spectra were collected at the Shanghai Synchrotron Radiation Facility (SSRF, China). The N K-edge XANES were obtained at the BL12B beamline of National Synchrotron Radiation Laboratory (NSRL, China) under a total electron yield (TEY) mode with vacuum better than 5 × 10^−7^ Pa. The magnetization has been studied by means of SQUID magnetometer. Magnetic and temperature-dependent magnetization has been measured by SQUID at a temperature range of 5 ∼ 400 K (500 Oe) and a magnetic field up to 4 T. The ICP-AES analysis was performed on Optima 7300 DV. For characterization by SEM, EDS, and XPS, the powder was dispersed in ethanol *via* ultrasonication to form a stable colloidal suspension. A drop of the suspension was then drop-cast onto a clean silicon wafer with a thermally grown SiO_2_ layer and allowed to dry under ambient conditions, forming a thin layer of nanosheets on the inert substrate for measurement.

## Author contributions

W. H. and C. W. conceived the experiments and supervised the project; W. H. and R. L. performed the TEM, SEM, EXAFS and XANES measurements; R. L. performed the magnetic measurements; C. W. and Y. Z. performed the DFT calculations; W. H., R. L., and Y. Z. analyzed the results; and W. H., C. W., and R. L. wrote the paper with comments from all authors.

## Conflicts of interest

There are no conflicts of interest to declare.

## Supplementary Material

RA-016-D5RA08017G-s001

## Data Availability

The data that support the findings of this study—including the raw experimental measurements (XRD, SEM, TEM, XPS, XANES, EXAFS, SQUID) along with computational details and results (DFT)—are available within the article and its supplementary information (SI). For phase identification *via* XRD, standard reference patterns from the International Centre for Diffraction Data (ICDD) database were used. Supplementary information: includes the structural model of the 2 × 2 × 1 Mo_2_N supercell (Fig. S1) and the calculated total energies for both ferromagnetic and antiferromagnetic configurations (Table S1). See DOI: https://doi.org/10.1039/d5ra08017g.
