# Supplementary material for: Molten salt-directed catalytic synthesis of room-temperature ferromagnetic transition-metal nitride nanosheets
Source: RSC Adv. 2026 Jan 19;16(5):4053–61. doi: 10.1039/d5ra08017g (PMC12814936; doi:10.1039/d5ra08017g)
Supplement: RA-016-D5RA08017G-s001 [file RA-016-D5RA08017G-s001.pdf]

Supplementary Information (SI) for RSC Advances.
This journal is © The Royal Society of Chemistry 2026

**Supporting Information**

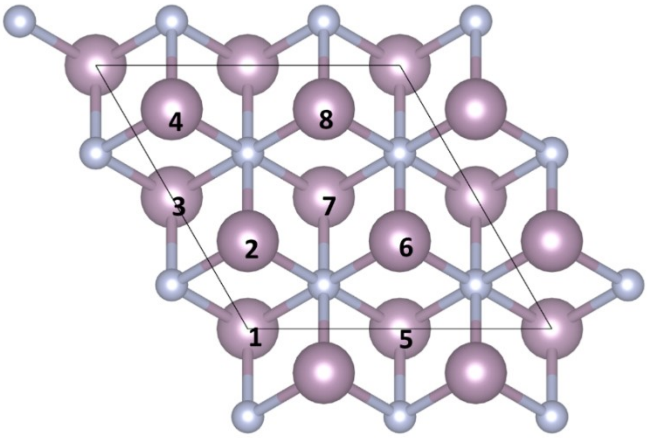


Figure S1. 2×2×1 $Mo_2N$ supercell model for calculation.

Table S1. Energy of various magnetic configurations

| No. | Energy (eV) | Magnetic moments of Mo atoms ($\mu_B$) | | | | | | | |
|---|---|---|---|---|---|---|---|---|---|
| | | 1 | 2 | 3 | 4 | 5 | 6 | 7 | 8 |
| 1 | -41102.54 (FM) | 0.3 | 0.3 | 0.3 | 0.3 | 0.3 | 0.3 | 0.3 | 0.3 |
| 2 | -41102.51 (AFM) | -0.03 | 0.03 | -0.02 | 0.03 | -0.02 | 0.02 | -0.02 | 0.03 |

We constructed a 2×2×1 supercell of monolayer γ-$Mo_2N$ (Figure S1), which contains 8 inequivalent Mo atoms. This supercell allows us to model a variety of magnetic configurations, including the ferromagnetic (FM) state and several antiferromagnetic (AFM) states by flipping the spins of different Mo sublattices. The calculated total energies for these configurations are summarized in the table S1. The key finding is that the FM state has the lowest energy, confirming it as the magnetic ground state, which is fully consistent with our experimental observations. Furthermore, The exchange energy (J) was fitted from the DFT-calculated energy of various magnetic configurations. And the obtained J values was 62 meV.
